# Research on dynamic characteristics and structural optimization of porous gas bearings in linear compressors

**DOI:** 10.1038/s41598-023-43818-z

**Published:** 2023-10-02

**Authors:** Jiangang Li, Jianjun Wu, Jingdao Fan, Xin Wang, Zhongjie Gao

**Affiliations:** 1 School of Mechanical Engineering, Liaoning Technical University, Fuxin, 123000 Liaoning China; 2 Research Institute of Technology and Equipment for the Exploitation and Utilization of Mineral Resources, Fuxin, 123000 Liaoning China; 3 Shaanxi Yanchang Petroleum (Group) Co., Ltd., Xi’an, 710065 Shaanxi China

**Keywords:** Mechanical engineering, Fluid dynamics

## Abstract

In order to study the influence of structural parameters of porous gas bearing and operating parameters of linear compressor on the static and dynamic performance of porous gas bearing, based on gas lubrication theory, Darcy's law and Reynolds equation, the mathematical model and simulation model of porous gas bearing of linear compressor are derived and established. The static and dynamic characteristics of the porous gas bearing of the linear compressor are studied by using Fluent software simulation. According to the simulation results, the effects of inlet pressure, porous material thickness and gas gap on the gas consumption and bearing capacity of the porous gas bearing under different eccentricities are analyzed. The results show that the higher the inlet pressure is, the larger the gas consumption and bearing capacity; the thicker the porous material is, the smaller the gas consumption and the larger the bearing capacity, the thicker the gas gap is, the larger the gas consumption and the smaller the bearing capacity. On the basis of simulation research, considering the difficulties of processing and assembly, multi-objective optimization of porous gas bearings is carried out based on response surface methodology. Taking the bearing capacity and gas consumption as the objective functions, the intake pressure is set between 0.3 and 0.5 MPa, the thickness of porous materials is set between 3 and 5 mm, and the thickness of gas gaps is set between 10 and 20 μm. While ensuring the stable operation of the linear compressor, the optimal combination of design parameters is provided for the optimal design of gas bearings used in linear compressors.

## Introduction

In a conventional compressor, the piston is driven by a crank linkage mechanism and lubricated with lubricating oil. Based on the conventional compressor, the linear compressor eliminates the crank drive mechanism and converts the rotational motion of the rotary motor into a reciprocating linear motion of the piston within the compression chamber. In the reciprocating linear motion of the piston, it is constantly subjected to lateral forces that push the piston against the cylinder, increasing frictional losses and noise vibration. To reduce frictional losses, either plate spring technology or gas bearing technology is generally used between the piston and the cylinder to ensure non-contact operation between the cylinder and the piston^1,2^. Since gas bearings have the advantages of low friction, no contamination, high rotational accuracy, high pressure and high and low temperature resistance compared to plate springs, they are more suitable for the working scenario of linear compressors and can further expand the application area of linear compressors^3,4^.

Sunpower achieved non-contact operation between cylinder and piston by using hydrostatic gas bearing technology in a linear compressor, reducing lateral forces on the piston and improving the efficiency and service life of the Stirling refrigerator^5^. Kuo et al. designed and analyzed gas bearings for an L-3 Stirling cycle refrigerator and verified the feasibility of gas bearing technology for application in a linear compressor^6^. The R&D department of Embraco and the Laboratory of Emerging Technologies Research at the Federal University of Santa Catarina, Brazil, jointly studied the equilibrium conditions of a small-bore throttled gas-lubricated piston for a linear compressor and analyzed the influence of some design parameters on the equilibrium conditions of the piston, such as gas gap thickness, throttled small-bore diameter, and throttled small-bore distribution position^7^.

The 16th Research Institute of China Electronics Technology Group Corporation has successfully developed a small Stirling refrigerator with gas bearings by using gas bearing technology. Huiming Zou et al. applied the porous gas bearing to a linear compressor, and analyzed the effects of the structural parameters of the porous bearing and the operating parameters of the compressor on the gas consumption and rate of the porous gas bearing using Fluent software. The accuracy of the simulation model was verified through experimental tests. Many domestic scholars have studied the operating characteristics of linear compressors using CFD technology^8–11^ and conducted static and dynamic characterization on the effects of gas bearing structural parameters on the load carrying capacity and gas consumption mass flow rate of gas bearings. This has a guiding significance for the design of structural parameters of gas bearings in linear compressors.

At present, linear compressors mostly use small-hole throttling gas bearings. Compared with the small-hole throttling gas bearing, the porous gas bearing increases the gas supply area and makes the pressure distribution in the throttled gas gap more uniform, which effectively improves the stability and performance of the compressor. This paper mainly focuses on the working characteristics of linear compressors and simulates the static and dynamic characteristics of porous gas bearings by using Fluent software^12^, which provides a reference for the optimization of porous gas bearings for linear compressors.

## Working principle and mathematical modeling of porous gas bearing

### Multi-hole bearing model and parameters

**Figure 1:**
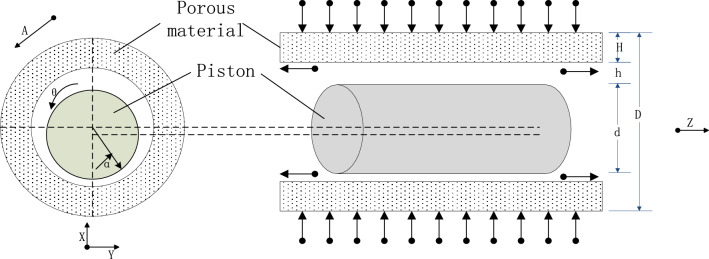
Structure of porous gas bearing.

Porous gas bearing is a layer of porous media material added to the inner layer of the bushing. The bearing material selected in this article is a carbon fiber reinforced carbon matrix composite material developed by the German Aerospace Center. This material not only has extreme temperature resistance, high pressure resistance, and friction resistance, but also has many advantages such as low density, high strength, high modulus, thermal expansion performance, and extremely low thermal conductivity. The permeability of porous materials is 5.57 × 10^−14^ m^2^, with a porosity of 0.18. Its main feature is that there are many micro-pores inside. The schematic diagram of the structure of the porous gas bearing and the cylindrical coordinate system used is shown in Fig. 1. The structural dimensions of the porous piston cylinder are shown in Table 1. The main feature of hydrostatic gas lubrication is the introduction of high pressure gas in the exhaust chamber, which is supplied from the cylindrical outer surface of the porous material. The gas is throttled through the resistance of the pores inside the porous material, and then flows into the gap between the cylinder and the piston to form a gas film between the cylinder and the piston. Under the condition that the piston has a certain eccentricity, a load-bearing mechanism is established for the cylinder piston. Finally, from both sides of the gas film flow into the compression and discharge chambers of the linear compressor respectively. Therefore, the flow of gas in the bearing is divided into two main parts, one is the flow of gas in the porous media material and the other is the flow of gas in the bearing gap^13–18^.

**Table 1:** Structural dimensions of porous gas bearings.

Cylinder length*/*mm	Piston diameter*/*mm	Porous material thickness*/*mm	Porous material length*/*mm	Air gap thickness/$\mathrm{\mu m}$
50	10	5	50	20

### Solution of gas pressure distribution equation

#### Pressure distribution equation of inside porous material

The gas in porous materials is mainly viscous flow, when the viscous gas flows through the porous material with a certain pressure difference, the gas motion must satisfy Darcy’s law, that is, the relationship between the gas velocity and the gas pressure gradient in all directions of the porous medium is^19–22^:

1 $$ V = - \frac{\left\| \phi \right\|}{\eta }\Delta p^{\prime} $$

where $V$ is the gas velocity inside the porous material,$\phi$ is the internal viscous permeability of the porous material, $\eta$ is the dynamic viscosity coefficient of the gas, and ${p}^{\prime}$ is the internal gas pressure of the porous material.

In the cylindrical coordinate system, the velocity of the gas moving through the porous material can be expressed as

2 $$ V = - \frac{\left| \phi \right|}{\eta }\left( {\frac{{\partial p^{\prime}}}{\partial x}\vec{i} + \frac{{\partial p^{\prime}}}{\partial y}\vec{j} + \frac{{\partial p^{\prime}}}{\partial z}\vec{k}} \right) $$

The velocity of the gas movement in all directions within the porous material is

3 $$ \left. {\begin{array}{*{20}c} {u^{\prime} = - \frac{{\phi_{x} }}{\eta }\frac{{\partial p^{\prime}}}{\partial x}} \\ {v^{\prime} = - \frac{{\phi_{y} }}{\eta }\frac{{\partial p^{\prime}}}{\partial y}} \\ {w^{\prime} = - \frac{{\phi_{z} }}{\eta }\frac{{\partial p^{\prime}}}{\partial z}} \\ \end{array} } \right\} $$

where u′ is the velocity component of the gas in the x-direction in the porous medium, v′ is the velocity component of the gas in the y-direction in the porous medium, and z′ is the velocity component of the gas in the z-direction in the porous medium.

In the column coordinate system, the continuity equation for the flow of a gas in a porous medium is

4 $$ \frac{{\partial \left( {\rho \phi } \right)}}{\partial t} + \frac{{\partial \rho {\text{u}}^{\prime } }}{\partial x} + \frac{{\partial \rho v^{\prime } }}{\partial y} + \frac{{\partial \rho w^{\prime } }}{\partial z} = 0 $$

In gas lubrication, the motion of the gas can be approximated as an isothermal process. For an isothermal gas motion process, the equation of state of the gas is

5 $$ \frac{{{\text{p}}^{\prime } }}{\rho } = \frac{{p_{a} }}{{\rho_{a} }} $$

Substituting the gas velocity components in each direction within the porous material into Eq. (4), the equation for the gas pressure distribution within the porous material is obtained according to Eq. (5):

6 $$ \frac{{\phi_{x} }}{\eta }\frac{{\partial^{2} p^{\prime 2} }}{{\partial x^{2} }} + \frac{{\phi_{y} }}{\eta }\frac{{\partial^{2} p^{{\prime}{2}} }}{{\partial y^{2} }} + \frac{{\phi_{z} }}{\eta }\frac{{\partial^{2} p^{{\prime}{2}} }}{{\partial z^{2} }} = 0 $$

In the obtained pressure distribution equation, each parameter has a different order of magnitude. In order to distinguish and compare these parameters, the terms with greater influence are retained, while those with less influence are neglected, in order to simplify the basic equation and make it dimensionless:

7 $$ \frac{{H^{2} }}{{B^{2} }}\phi_{{\text{x}}} \frac{{\partial^{2} \overline{p}^{{\prime}{2}} }}{{\partial \overline{x}^{2} }} + \phi_{y} \frac{{\partial^{2} \overline{p}^{{\prime}{2}} }}{{\partial \overline{y}^{2} }} + \frac{{H^{2} }}{{B^{2} }}\phi_{Z} \frac{{\partial^{2} \overline{p}^{{\prime}{2}} }}{{\partial \overline{z}^{2} }} = 0 $$

Where $\overline{x}$ is the dimensionless coordinate in x-direction,$\overline{x} = \frac{x}{B}$, B is the characteristic length of the bearing in x-direction;$\overline{y}$ is the dimensionless coordinate in y-direction,$\overline{y} = \frac{y}{H}$, H is the thickness of the porous material;$\overline{z}$ is the dimensionless coordinate in z-direction,$\overline{z} = \frac{z}{L}$, and L is the characteristic length of the bearing in z-direction;$\overline{p}^{\prime}$ is the dimensionless pressure distribution of the porous material,$\overline{p}^{\prime} = \frac{{p^{\prime}}}{{p_{0} }}$.

#### Pressure distribution equation of gas gap

The gas flow in the bearing gap must satisfy mass conservation, i.e. the difference between the inflow and outflow mass flow rates of the microelement should be equal to the inflow mass flow rate of the microelement^23–27^.

According to the law of conservation of mass, the continuity equation of the gas can be rewritten as

8 $$ \frac{\partial }{\partial x}\int_{0}^{h} {\rho udy} + \frac{\partial }{\partial z}\int_{0}^{h} {\rho wdy} = \rho \tilde{v} $$

where $\tilde{v}$ is the velocity of the gas from the porous medium into the intermembrane space of the gas,$\tilde{v} = \left. {v^{\prime}} \right|_{y = 0}$.

After neglecting the volume forces, the N–S equation can be simplified as

9 $$ \left. {\begin{array}{*{20}c} {\frac{\partial p}{{\partial x}} = \eta \frac{{\partial^{2} u}}{{\partial y^{2} }}} \\ {\frac{\partial p}{{\partial y}} = 0} \\ {\frac{\partial p}{{\partial z}} = \eta \frac{{\partial^{2} w}}{{\partial z^{2} }}} \\ \end{array} } \right\} $$

where *p* represents the gas pressure in the bearing gap.

When the gas enters the gas intermembrane space from the porous media region, the discontinuous gas velocity at the interface between the two will lead to velocity slip. According to the Beavers-Joseph model, the boundary slip condition can be obtained as follows:

10 $$ \left. {\begin{array}{*{20}c} {\left. {\frac{\partial u}{{\partial y}}} \right|_{y = 0} = \frac{\alpha }{{\phi_{x}^{1/2} }}\left( {u_{B} - u^{\prime}} \right) = \frac{\alpha }{{\phi_{x}^{1/2} }}u\left( {x,0,z} \right)} \\ {\frac{\partial v}{{\partial y}} = 0} \\ {\left. {\frac{\partial w}{{\partial y}}} \right|_{y = 0} = \frac{\alpha }{{\phi_{x}^{1/2} }}\left( {w_{B} - w_{z}^{\prime } } \right) = \frac{\alpha }{{\phi_{x}^{1/2} }}w\left( {x,0,z} \right)} \\ \end{array} } \right\} $$

where $\alpha$ is the velocity slip parameter related to porous materials, typically ranging from 0.01 to 4.0.

At the non-permeable surface ($y = h$), when the bearing velocity is $v = 0$, there are $u = 0$,$w = \omega$ and according to the simplified N–S equation and the boundary slip condition, we can get

11 $$ \left. {\begin{array}{*{20}c} {u = \frac{1}{2\eta }\frac{\partial p}{{\partial x}}\left( {y + \frac{{\zeta_{1} }}{3}h} \right)\left( {y - h} \right)} \\ {v = - \frac{{\phi_{y} }}{\eta }\frac{{\partial p^{\prime}}}{\partial y}} \\ {w = \frac{1}{2\eta }\frac{\partial p}{{\partial z}}\left( {y + \frac{{\zeta_{1} }}{3}h} \right)\left( {y - h} \right) + \frac{{2\zeta_{2} }}{h}\omega \left( {y - h} \right) + \omega } \\ \end{array} } \right\} $$

Where $$\zeta_{1}$$ is the velocity slip coefficient,$$\zeta_{1} = \frac{{3\phi^{{{\raise0.7ex\hbox{$1$} \!\mathord{\left/ {\vphantom {1 2}}\right.\kern-0pt} \!\lower0.7ex\hbox{$2$}}}} /\alpha }}{{\phi^{{{\raise0.7ex\hbox{$1$} \!\mathord{\left/ {\vphantom {1 2}}\right.\kern-0pt} \!\lower0.7ex\hbox{$2$}}}} /\alpha + h}}$$,$$\zeta_{2}$$ is the velocity slip coefficient, and $$\zeta_{2} = \frac{h}{{2\left( {\phi^{{{\raise0.7ex\hbox{$1$} \!\mathord{\left/ {\vphantom {1 2}}\right.\kern-0pt} \!\lower0.7ex\hbox{$2$}}}} /\alpha + h} \right)}}$$ and $$h$$ is the air film thickness.

Substituting Eq. (11) into the continuity equation and using the relation $p\frac{\partial p}{{\partial \xi }} = \frac{1}{2}\frac{{\partial p^{2} }}{\partial \xi }$ based on the gas equation of state, the equation for the gas pressure distribution in the space between the gas membranes is obtained as follows

12 $$ \begin{gathered} \frac{\partial }{\partial x}\left[ {h^{3} \left( {1 + \zeta_{1} } \right)\frac{{\partial p^{2} }}{\partial x}} \right] + \frac{\partial }{\partial z}\left[ {h^{3} \left( {1 + \zeta_{1} } \right)\frac{{\partial p^{2} }}{\partial z}} \right] + 12\eta p\tilde{v} \hfill \\ = 12\eta u_{2} \frac{\partial }{\partial x}\left[ {ph\left( {1 - \zeta_{2} } \right)} \right] + 12\eta w_{2} \frac{\partial }{\partial z}\left[ {ph\left( {1 - \zeta_{2} } \right)} \right] \hfill \\ \end{gathered} $$

Make it dimensionless:

13 $$ \begin{gathered} \frac{\partial }{{\partial \overline{x}}}\left[ {\overline{h}^{3} \left( {1 + \zeta_{1} } \right)\frac{{\partial \overline{p}^{2} }}{{\partial \overline{x}}}} \right] + \frac{\partial }{{\partial \overline{z}}}\left[ {\overline{h}^{3} \left( {1 + \zeta_{1} } \right)\frac{{\partial \overline{p}^{2} }}{{\partial \overline{z}^{2} }}} \right] + \overline{Q} \hfill \\ = \lambda \overline{u}\frac{\partial }{{\partial \overline{x}}}\left[ {\overline{p}\overline{h}\left( {1 - \zeta_{2} } \right)} \right] + \lambda \overline{w}\frac{\partial }{\partial z}\left[ {\overline{p}\overline{h}\left( {1 - \zeta_{2} } \right)} \right] \hfill \\ \end{gathered} $$

Where $\overline{x}$ is the dimensionless coordinate in x-direction,$\overline{x} = \frac{x}{l}$,$l$ is the reference quantity in the x direction;$\overline{z}$ is the dimensionless coordinate in z-direction,$\overline{z} = \frac{z}{l}$, $l$ is the reference quantity in the z direction;$\overline{p}^{\prime}$ is dimensionless pressure in the gas film gap, $\overline{p}^{\prime} = \frac{{p^{\prime}}}{{p_{0} }}$; $\overline{h}$ is dimensionless gas film thickness, $h = \frac{h}{{h_{0} }} = 1 - \varepsilon \cos \left( {\theta - \phi } \right)$; $\lambda$ is number of dimensionless bearings, $\lambda = \frac{24\eta Vl}{{h_{0}^{2} p_{0} }}$; $\overline{Q}$ is the dimensionless mass flow factor of the inflow gas,$\overline{Q} = \frac{{24\eta l^{2} }}{{h_{0}^{3} p_{0}^{2} }}p\tilde{v}$.

Substitute the velocity equations of gas in various directions in porous materials into $\overline{Q}$ to obtain:

14 $$ \overline{Q} = \frac{{24\eta l^{2} }}{{h_{0}^{3} p_{0}^{2} }}p\tilde{v} = \left. { - \frac{{12\phi_{y} l^{2} }}{{h_{0}^{3} p_{0}^{2} }}} \right|_{y = 0} = - \frac{{12\phi_{y} l^{2} }}{{h_{0}^{3} p_{0}^{2} }}\frac{{p^{2} - p_{0}^{2} }}{H} $$

### Fluent pre-processing

**Figure 2:**
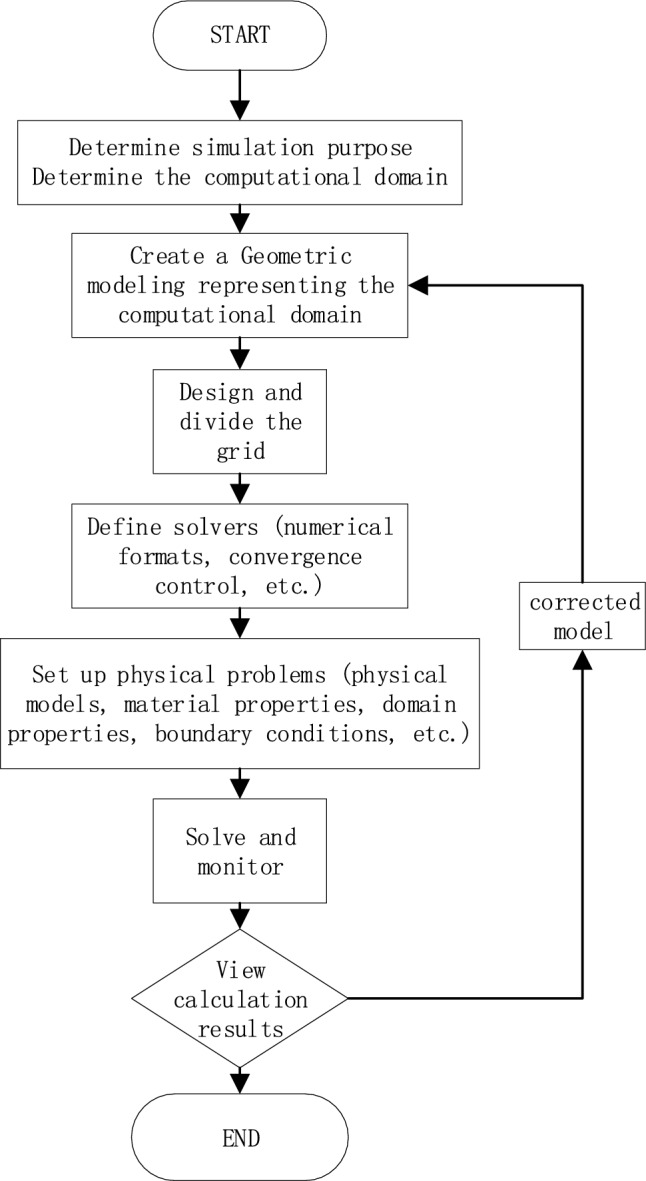
Flow chart of the calculation process.

Workbench is a window software developed by ANSYS, integrated by structural mechanics module and fluid mechanics simulation module. Fluent module is widely used in mechanical, aerodynamic noise, multiphase flow system, etc. due to its rich physical model. Fluent simulation consists of three major parts: pre-processing (model building, meshing, boundary definition), solver (porous material properties, fluid properties, boundary conditions, dynamic mesh, etc.), and post-processing (pressure clouds, velocity clouds, load carrying capacity, etc.). Figure 2 shows the flow chart of Fluent simulation calculation process.

**Figure 3:**
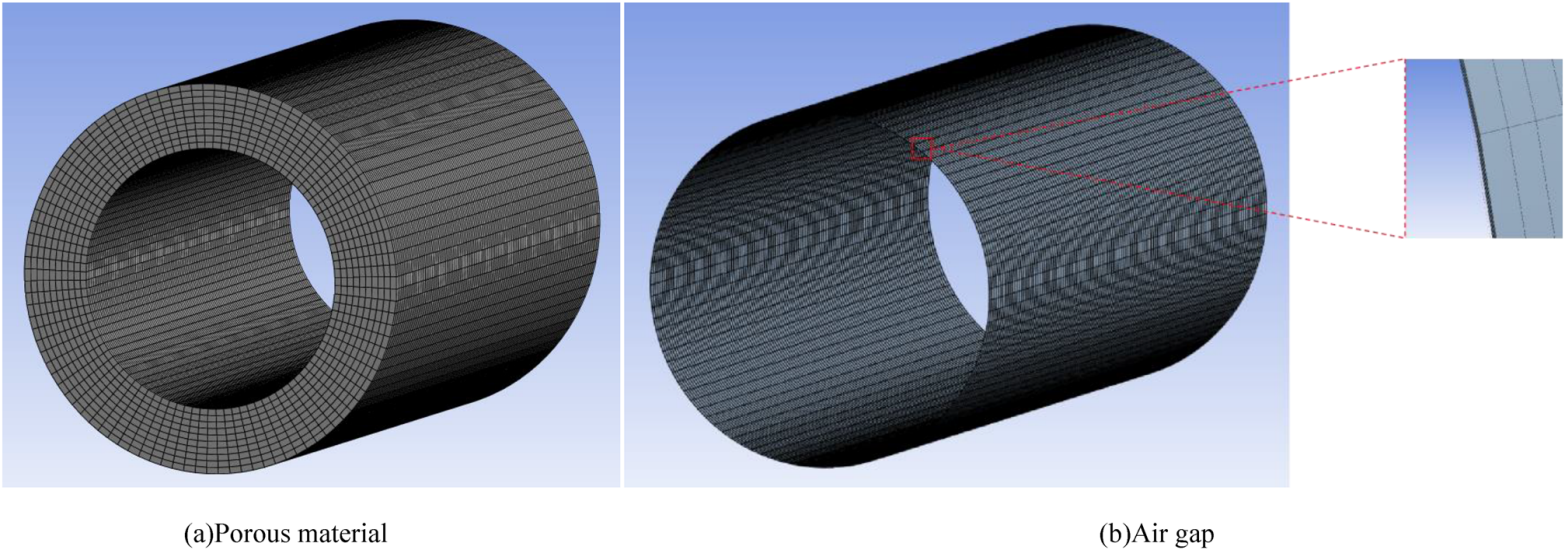
Mesh generation for (**a**) porous material and (**b**) air gap.

First, Solidworks was used to model the porous gas bearing. Since the thickness of the air gap is in the micron level and the difference between axial and radial dimensions is thousands of times, it is difficult to mesh and highly prone to errors. Meanwhile, the quality of the mesh directly affects the accuracy and efficiency of the simulation^28–30^. After continuous attempts, while ensuring calculation accuracy and speed, the inner and outer diameters of the porous gas radial bearing, as well as the circumference of the air gap inner and outer diameters, are taken as equal grid numbers ${n}_{1}={n}_{2}={n}_{3}={n}_{4}=200$, and the length direction of the bearing is taken as ${n}_{5}=100$. And the block meshing method is adopted to use regular rectangular mesh. In addition, the number of mesh nodes in the thickness of the porous material and the thickness direction of the air gap depends on different cases. For example, when the porous material is $3\, \mathrm{mm}$ , take ${n}_{6}=30$ . The volume mesh is adopted as a positive hexahedral mesh with a mesh number of about 500,000. The mesh division is shown in Fig. 3, where Fig. 3a shows the mesh division of the porous material and Fig. 3b shows the mesh division of the bearing air gap.

After the mesh delineation is completed, the 3D double precision calculation method is selected when the file is imported into Fluent for steady-state calculations. Before entering Fluent for calculation, a mesh quality check is required to ensure that the mesh can be used for the calculation. The implicit pressure-based solver is selected, the energy equation and turbulence model is enabled for its physical calculation model, the physical parameters of the flowing gas and the porous area is then defined, and the viscous drag coefficient and inertial drag coefficient of the porous material are set. In the simulation of static characteristics, steady-state calculation is used, and the boundary conditions of inlet and outlet are set as pressure inlet and outlet boundary conditions. The pressure–velocity coupling method is simple, the pressure discretization method is standard, and the other parameters discretization method is second-order upwind. If the parameters set by default during the calculation cannot meets the stability requirements, and the residual curves oscillate and diverge, the convergence of the calculation can be accelerated by reducing the relaxation factor.

## Static characteristics analysis of porous gas bearing

The load carrying capacity and gas consumption of the bearing are used to describe the static characteristics of a porous aerostatic bearing. The load carrying capacity is obtained by integrating the fluid pressure over the fluid lubrication area, and the gas consumption of the bearing is obtained by calculating the internal velocity of flow through the bearing gap. Factors affecting the load carrying capacity and gas consumption of the gas bearing include the inlet pressure of the gas bearing ${P}_{0}$ the thickness of the porous material $H$ and the average air gap thickness $h$. Eccentricity $\upvarepsilon$ equals the ratio between eccentricity of the piston and the porous sleeve to the thickness of the air gap.

### Effect of air inlet pressure

The variation of static characteristics of the porous gas bearing at different inlet pressures and eccentricities is simulated and calculated with the porous material thickness $H=5\, \mathrm{mm}$ and average air gap thickness $h=20\,\upmu$m. The discharge pressure of the linear compressor used in this study is generally between 0.2 and 0.7 MPa. Therefore, when studying the static characteristics of porous gas bearings, inlet pressures of 0.6 MPa, 0.5 MPa, 0.4 MPa, 0.3 MPa, and 0.2 MPa are selected.

**Figure 4:**
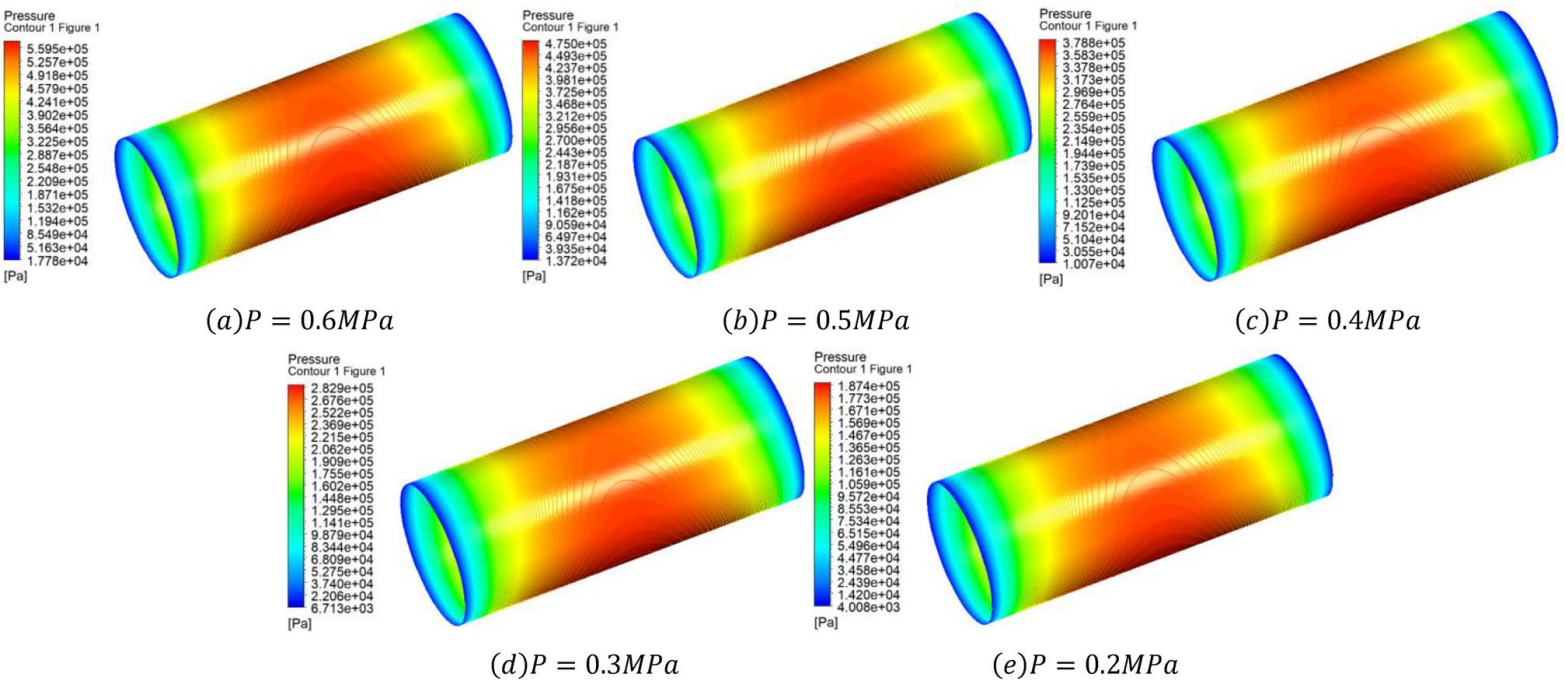
When the thickness of porous material $H=5\, \mathrm{mm}$, the average air gap thickness $h=20\, \mathrm{\mu m}$, eccentricity $\upvarepsilon =0.3$, the air gap pressure cloud at different inlet pressure.

Figure 4 shows when the thickness of porous material $H=5\, \mathrm{mm}$, the average air gap thickness $h=20\, \mathrm{\mu m}$ , eccentricity $\upvarepsilon =0.3$, the air gap pressure cloud at different inlet pressure. As can be seen from the graph, the pressure drops after the gas passes through the porous medium and enters the bearing gap, and then flows axially to the ends, where the pressure gradually drops to standard atmospheric pressure. Due to the presence of eccentricity, the pressure on the shaft is high in the area with small bearing clearance and low in the area with large clearance.

**Figure 5:**
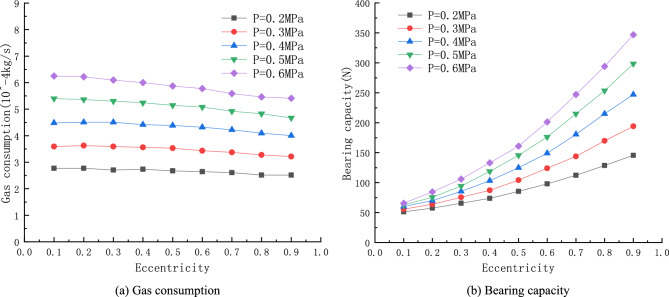
Curves of Gas consumption and Bearing capacity with inlet pressure.

As shown in Fig. 5a, when the thickness of the porous material and the average air gap thickness are constant, the gas-consuming mass flow rate increases with the increase of the supply pressure, and the change of the gas consuming mass flow rate is not greatly influenced by the eccentricity. For every increase in the inlet pressure with $0.1\, \mathrm{MPa}$ the maximum increase of gas consumption is about $20\%$. When the inlet pressure increases to $0.6\, \mathrm{MPa}$ the maximum gas consumption reaches ${6.3\times 10}^{-4}\, \mathrm{kg}/\mathrm{s}$. The increase in gas consumption is due to the increase in inlet pressure, which increases the pressure difference between the inlet and outlet air, which increases the velocity of the gas inlet and outlet air, which increases the mass flow rate of the consumed air.

As shown in Fig. 5b, when the thickness of porous material and the average air gap thickness are certain, the bearing capacity of porous gas bearing will increase to a certain extent as the inlet pressure increases in a certain range, and the larger the eccentricity of gas bearing under the same supply pressure the larger the bearing capacity of gas bearing. The maximum load capacity of porous gas bearing is $353\, N$, when the inlet pressure is $0.6\, \mathrm{MPa}$ and the eccentricity is 0.9. This is because the load carrying capacity comes from the pressure difference between the upper and lower part of the gas film. Thus when the gas supply pressure is increased, the highest pressure and high pressure area in the air gap also increases, the pressure difference between the upper and lower part of the gas film also becomes larger, so the bearing load carrying capacity also becomes larger. However, while increasing the inlet pressure to obtain a larger load capacity, it will lead to excessive gas consumption and increase the cost of the linear compressor usage.

### Effect of thickness of porous material

**Figure 6:**
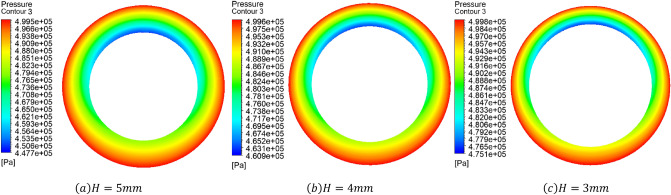
When the air supply pressure $P=0.5\, \mathrm{MPa}$, the average air gap thickness $h=20\, \mathrm{\mu m}$, eccentricity $\upvarepsilon =0.3$ The pressure drop generated when the gas flows through the porous material.

It is necessary to study the static performance of porous gas bearings with varied porous material thicknesses. The simulations were chosen to study the variation of static performance of porous gas bearing with the porous material thicknesses at $H=5\, \mathrm{mm}$, $H=4\, \mathrm{mm}$ and $H=3\, \mathrm{mm}$ when the inlet pressure $P=0.5\, \mathrm{MPa}$ and the average air gap thickness $h=20\, \mathrm{\mu m}$. Figure 6 shows the pressure drop cloud map generated by gas flowing through porous materials with varied thicknesses. For every $1\, \mathrm{mm}$ decrease in thickness of porous materials, the pressure drop generated by gas flowing through porous materials decreases by $0.015\, \mathrm{MPa}$, indicating that the greater the thickness of porous materials, the more obvious their throttling effect.

**Figure 7:**
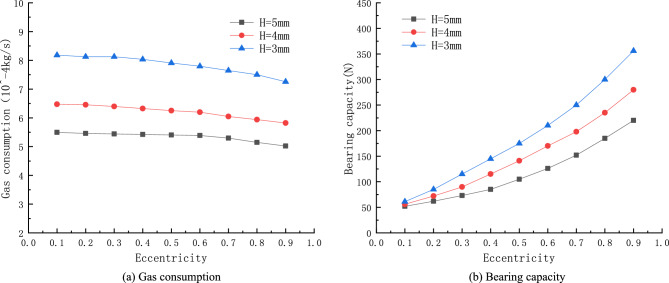
Curves of Gas consumption and Bearing capacity with thickness of porous material.

As shown in Fig. 7a, as the thickness of porous materials increases, the gas consumption mass flow rate of the bearing will decrease, and the trend of gas consumption reduction will become more significant as the increase of eccentricity. When the thickness of the porous material is $5\, \mathrm{mm}$, the gas consumption of the bearing remains around ${5.5\times 10}^{-4}\, \mathrm{kg}/\mathrm{s}$. When the thickness of the porous material is $4\, \mathrm{mm}$, the gas consumption of the bearing is guaranteed to be around ${6.5\times 10}^{-4}\, \mathrm{kg}/\mathrm{s}$. When the thickness of the porous material is $3\, \mathrm{mm}$, the gas consumption of the bearing remains around ${8.1\times 10}^{-4}\, \mathrm{kg}/\mathrm{s}$. This is because the increase in the thickness of the porous material will increase the pressure drop generated by the gas flowing through the porous material, and the pressure distribution in the gas gap will become more uniform, and the gas consumption mass flow rate will also be reduced accordingly.

As shown in Fig. 7b, the thicker the porous material, the greater the pressure drop generated by gas flowing through the porous material, resulting in a decrease in pressure in the air gap and a corresponding decrease in load-bearing capacity. When the thickness of porous material in porous gas bearings is $3\, \mathrm{mm}$ and the eccentricity is 0.9, the maximum bearing capacity is $364\, N$. However, in practical situation, it is not so simple that the smaller the thickness of porous materials, the better. The smaller the thickness of the material, the more difficult it is to process, and the pressure drop generated by gas flowing through porous materials will decrease. The throttling surface of porous materials may undergo deformation under high gas supply pressure. Since the gas film thickness of gas bearings themselves is on micrometer scale, any small deformation may cause instability working state or even damage on the gas bearing. Therefore, it can be seen that from the perspective of load-bearing capacity and dynamic stability, the thickness of porous materials should be minimized as much as possible while ensuring the stable operation of porous gas bearings.

### Effect of average air film thickness

**Figure 8:**
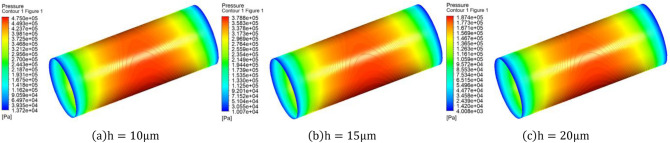
When the air supply pressure $P=0.5\, \mathrm{MPa}$, porous material thickness $H=5\, \mathrm{mm}$ and eccentricity $\upvarepsilon =0.3$, air gap pressure clouds under different air gap thickness.

The average gas film thickness is generally the thickness of the gas film when the porous material and the piston are partially coaxial (eccentricity $\mathrm{\upepsilon }=0$), and the gas gap of the gas bearing is generally taken to be between $\frac{1}{1000}\mathrm{and }1/5000$ of its diameter. The effect of the average gas film thickness on the static performance of the porous gas bearing is simulated at $h=10\, \mathrm{\mu m}$, $h=15\, \mathrm{\mu m}$ and $h=20\, \mathrm{\mu m}$ with the inlet pressure $P=0.5\, \mathrm{MPa}$ and porous material thickness $H=5\, \mathrm{mm}$. Figure 8 shows the gas gap pressure maps for different air gap thicknesses. It is obvious that the high pressure distribution on the piston is wider and the pressure magnitude is larger when the air gap thickness is smaller.

**Figure 9:**
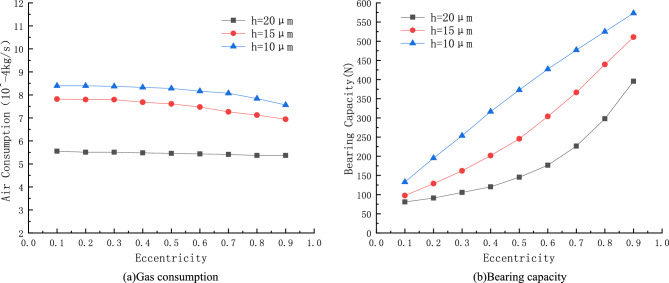
Curves of Gas consumption and bearing capacity with air gap thickness.

From Fig. 9a, it can be observed that the mass flow rate of porous gas bearings increases with the thickening of the average air gap, and as the gas film thickness increases, the trend of that mass flow rate decreasing with the decrease of eccentricity is gradually evident. When the air gap thickness is $h=20\, \mathrm{\mu m}$, the gas consumption remains at ${5.5\times 10}^{-4}\, \mathrm{kg}/\mathrm{s}$. When the air gap thickness is $h=15\, \mathrm{\mu m}$, the gas consumption remains at ${4.9\times 10}^{-4}\, \mathrm{kg}/\mathrm{s}$. When the air gap thickness is $h=10\, \mathrm{\mu m}$, the gas consumption remains at ${3.1\times 10}^{-4}\, \mathrm{kg}/\mathrm{s}$. The main reason why the change in air gap thickness has an impact on gas consumption is that thickening the air gap increases the gas outlet area and also increases the leakage loss of the compressor, resulting in an increase in the mass flow rate of the outlets on both sides of the gas film, and thus increasing the gas consumption mass flow rate of the gas bearing.

From Fig. 9, it can be observed that the load-bearing capacity of porous gas bearings increases with the decrease of average gas film thickness. When the air gap thickness of the porous gas bearing is $10\, \mathrm{\mu m}$ and the eccentricity is 0.9, the maximum bearing capacity is 556 *N*. The selection of average gas film thickness should be determined based on the demand for bearing capacity and stiffness. Generally speaking, bearing capacity and stiffness increase with the decrease of gas film thickness. Excessive gas film thickness will lead to an increase in mass flow rate and a decrease in bearing capacity. Nevertheless, too small gas film thickness will increase the difficulty of machining and assembling for gas bearings.

## Dynamic characteristics analysis of porous gas bearing

**Figure 10:**
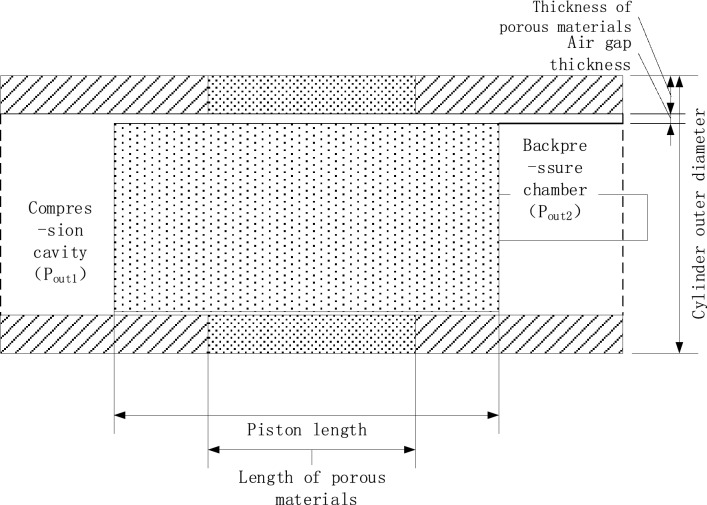
Cross-sectional view of a porous gas bearing piston cylinder.

When analyzing the dynamic characteristics of the porous gas bearing, the motion of the piston should be taken into account first. The internal structure of the compressor cylinder is shown in Fig. 10. During the operation of the compressor, the gas pressure in the compression chamber is constantly changing, resulting in a constant change in the gas outlet pressure P_out1_ at one end of the compression chamber. When the compressor is operating under ideal conditions, the piston makes a simple harmonic motion in the cylinder, and the simple harmonic motion of the piston causes the gas pressure in the compression chamber to show a corresponding sinusoidal change in one cycle. The outlet pressure can be approximated as

15 $$ P = - A\sin \left( {\omega t} \right) + \overline{P} $$

where *A* is the pressure amplitude, taken as *A* = *0.5 * MPa*.* $\upomega$ is the angular frequency, taken as $\upomega =100\uppi$. $\overline{P }$ is the average pressure of the compression chamber, set to $\overline{P }$=0.25 MPa. At this point, make ${P}_{out2}=0.2\, \mathrm{MPa}$, ${P}_{in}=0.3\, \mathrm{MPa}$, air gap thickness $h=20\, \mathrm{\mu m}$, and eccentricity $\upvarepsilon =0.3$. The non-stationary solver in Fluent is set up to analyze the dynamic characteristics of porous gas bearings, and the changes in bearing capacity and gas consumption mass flow rate within a complete cycle is expected to be obtained. In the non-stationary solution setting, 25 time steps are set within a period of $T=0.02\, \mathrm{s}$, with each time step iterating 350 times.

### Effect of dynamic pressure outlet on gas consumption

**Figure 11:**
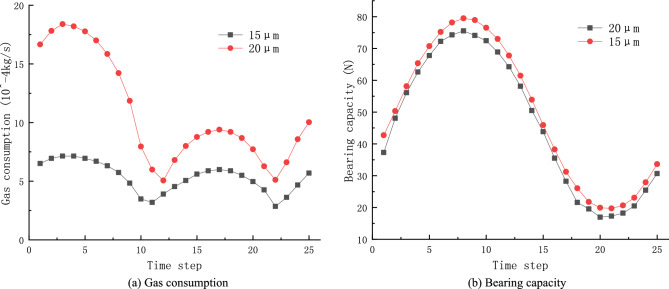
Effect of dynamic outlet pressure on Gas consumption and Bearing capacity.

Under static conditions, the outlet pressures on both sides of the gas film are constant values and the gas consumption is equal to the difference between the gas inlet and outlet mass flow rates. Under dynamic conditions, the periodic compression and expansion of the gas in the compression chamber forms a pressure wave due to the approximate simple harmonic motion of the piston, resulting in a corresponding periodic inflow or outflow of the gas in the compression chamber into or out of the gas gap. At this time, the pressure change at the pressure outlet end P_out1_ is the same as the pressure change in the compression chamber. When the gas flows into the gas film, there is only one pressure exit end P_out2_. At that point, the gas consumption of the porous gas bearing is determined by both the inlet end of the porous material and the pressure exit end P_out2_. Figure 11a shows the variation of gas consumption in one period when the air gap thickness $h=20\, \mathrm{\mu m}$ and $h=15\, \mathrm{\mu m}$. As can be seen from the figure, the gas consumption shows a periodic variation with the pressure change at the dynamic pressure outlet end. When the air film thickness increases, the gas consumption increases rapidly, which increases the leakage loss and reduces the efficiency of the linear compressor to some extent; the gas consumption is smaller when the air film gap is smaller. Therefore, when choosing the air film thickness, both the processing and assembly difficulty should be considered, and the power consumption loss caused when the air film gap is too large should also be considered.

### Effect of dynamic pressure outlet on bearing capacity

As shown in Fig. 11b, when the pressure at the dynamic pressure outlet varies as a sinusoidal function, the bearing capacity also shows a periodic variation, but the amplitude and duration of the first half-cycle and the second half-cycle are asymmetric. It can be seen from the graph that as the gas expands, the bearing capacity increases and lasts longer. While when the gas is compressed, the situation is the opposite. This is because when the piston does reciprocating linear motion in the cylinder, the pressure of the back pressure chamber gradually increases, the equilibrium position of the piston gradually deviates from the static equilibrium position, the cylinder gap volume increases, resulting in amplitude of the gas bearing force during the expansion and compression process. In addition, the stroke of the piston gradually decreases due to the influence of gas resistance, resulting in asymmetry of gas expansion and compression times. The figure also shows that when the gas film becomes smaller, the dynamic load-bearing capacity of the porous gas bearing becomes correspondingly larger, which is consistent with the static characteristics of the porous gas bearing.

## Optimal design of porous gas bearings based on response surface methodology

### Optimization of design methods

In the traditional optimization design, most designers use the design experiment or simulation analysis method, combined with their own experience, to provide several different gas bearing design schemes, and then according to the experiment or simulation results to choose the optimal scheme, to achieve the purpose of bearing optimization. This method takes a single parameter as an independent variable, ignoring the influence of the interaction of parameters on the performance of gas bearing. Response surface method is an optimization method combining experimental design and mathematical statistics, which can comprehensively consider the interaction between various parameters to comprehensively optimize the gas bearings. It performs continuous trials on the specified set of design points, solves the relationship between parameters and variables, builds response surfaces, and constructs global approximations of measured quantities in the design space. The response surface method is computationally simple and can fit complex response relationships by choosing a regression model with good robustness. Therefore, this method can overcome the disadvantages of the traditional optimization design and realize the true optimized design^22–31^.

There are many directions to optimize the performance of gas bearings, but most of them are to improve the bearing capacity $W$ and reduce the gas consumption $M$. Therefore, the optimized design model takes the inlet pressure $P$, the porous material thickness $H$ and the air gap thickness $h$ as the design variables, and takes the maximum bearing capacity W and the minimum gas consumption M as the objective functions of the optimized design:

16 $$ \begin{array}{*{20}c} {\max W = f_{1} \left( {H,h,P} \right)} \\ {\min M = f_{2} \left( {H,h,P} \right)} \\ \end{array} $$

There is a contradiction between those two objective functions. For example, reducing the air gap thickness will increase the bearing capacity and reduce the mass flow rate of gas consumption. Therefore, the weight of the objective function should be considered in the design process.

### Multi-objective optimization design process

The optimization process is as follows: Firstly, a three-dimensional flow field model is established to parameterize the thickness *H* and air gap *h* of the porous material. The parameters to be optimized are shown in Table 2. Then, divide the mesh and select the model in Fluent, set the boundary, parameterize the gas supply pressure *P*, calculate the static characteristics of the porous gas bearing, including the bearing capacity *W* and gas consumption *M*, and set them as the output parameter. After the confirmation that the results match with the actual situation, it turns to the response surface optimization module, the test points is designed, and the response surface is calculated. After ensuring the accuracy of the response surface, the best design parameters are selected.

**Table 2:** Parameters to be optimized.

Parameter name	Parameter symbols	Initial size	Maximum value	Lowest value
Intake pressure	P	5 MPa	6 MPa	2 MPa
Porous material thickness	H	5 mm	6 mm	3 mm
Air gap thickness	h	20 μm	20 μm	10 μm

### Analysis of optimization results

**Figure 12:**
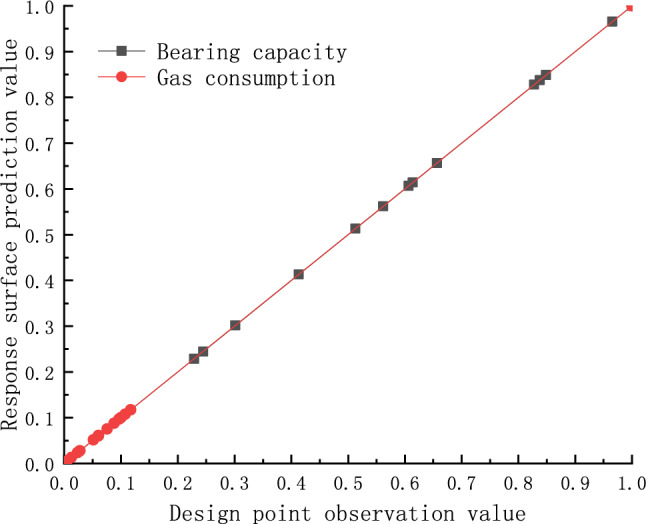
Fitting graph.

The sample was calculated using the experiments design approach of Central Integrated Design and then the sample data were fitted using the Kriging response surface model. The fit plots are shown in Fig. 12 below. It is obvious in the Fig. 12 that the observed values calculated from the design points are highly consistent with the predicted values fitted using the response surface. This result verified the reliability of the response surface model.

**Figure 13:**
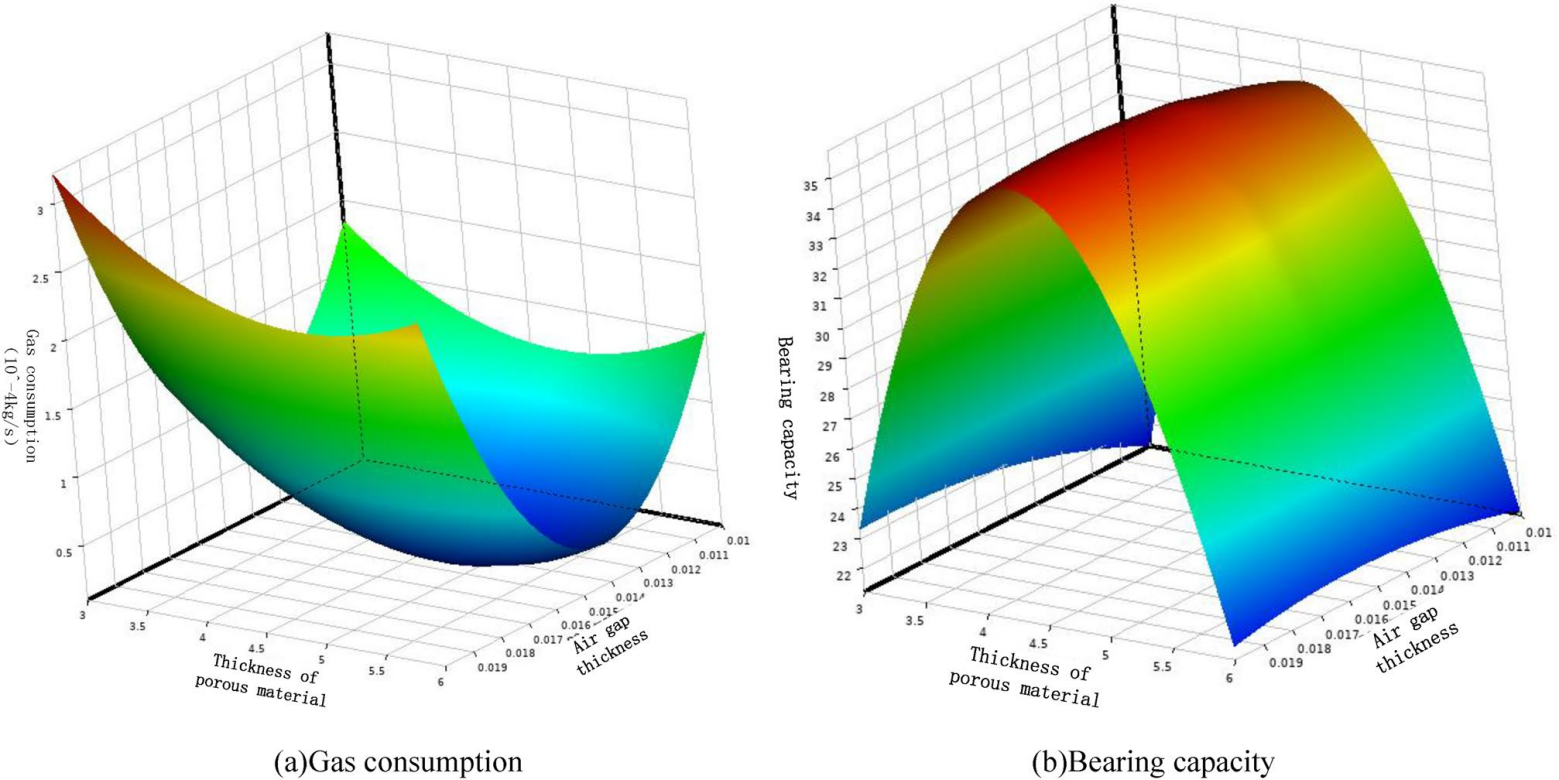
Influence of bearing structure parameters on Gas consumption and Bearing capacity.

The Fig. 13 shows the response surfaces of the influence of structural parameters on bearing capacity and gas consumption. Based on the response surface, it can be determined that the optimal thickness of the porous material is around 4.5 mm and the optimal air gap thickness is around $15\, \mathrm{\mu m}$. When the inlet pressure is 0.4 MPa, the maximum bearing capacity can reach around 37 *N* and the minimum gas consumption is around ${1.91\times 10}^{-4}\, \mathrm{kg}/\mathrm{s}$.

### Validation of optimized results

Based on the optimized structural parameters, a new simulation model was developed and calculated using the same boundary conditions as before. Finally, the calculated results were compared with the predicted results of the response surface, as shown in the following table.

As can be seen from Table 3, the predicted value and calculated results of the response surface model are very close, with only small errors, which further shows that the prediction accuracy of the response surface is good and can accurately reflect the actual output results of different parameter combinations.

**Table 3:** Validation of Optimized Results.

Parameter name	Optimization points	Calculation points	Error
Bearing capacity	36.83 N	34.45 N	6.46%
Gas consumption	1.91 × 10^–4^ kg/s	1.81 × 10^–4^ kg/s	4.73%

## Conclusion

In this paper, the structural parameters of porous gas bearings and the influence of the operating parameters of linear compressors on the static and dynamic performances of porous gas bearing around the porous gas bearing for linear compressors, and optimize the porous gas bearings considering the retrofitting process. The specific conclusions are as follows:

1. Based on gas lubrication theory, Dracy’s law and Reynolds equation, the gas pressure distribution equation for gas flow in porous materials and air gaps was derived and established, and the Fluent simulation model was built.
2. Through Fluent simulation, the effects of inlet pressure, porous material thickness and air gap thickness on gas consumption and bearing capacity were studied. The results show that the larger the inlet pressure is, the larger the gas consumption and bearing capacity is; the larger the thickness of porous material is, the smaller the gas consumption and the larger the bearing capacity is; the larger the thickness of air gap is, the larger the gas consumption and the smaller the bearing capacity is.
3. Compilation of pressure fluctuation function and piston periodic velocity function at the outlet of the compression chamber side of the gas membrane using UDF. Based on a static characteristics study, the simulation of the dynamic characteristics of the porous gas bearing is performed.
4. By establishing a response surface model, a multi-objective optimization is performed on the parameters that need to be modified to find the optimal solution. The optimized model is compared with the original model. The results show that when the thickness of the porous material is around 4.5 mm, the air gap thickness is around $15\, \mathrm{\mu m}$, and the inlet pressure is around 0.4 MPa, the maximum bearing capacity can reach around 37 *N*, and the minimum gas consumption is around ${1.91\times 10}^{-4}\, \mathrm{kg}/\mathrm{s}$.

## Data Availability

The datasets generated and analyzed during this study have not been made publicly available because the research in this paper relies heavily on a major project that has not yet been completed to conduct the study, and therefore the relevant research data are not yet publicly available. However, it can be obtained from the corresponding author upon reasonable request.
